# Nonmelanoma Skin Cancer at Critical Facial Sites: Results and Strategies of the Surgical Treatment of 102 Patients

**DOI:** 10.1155/2019/4798510

**Published:** 2019-06-26

**Authors:** Carlos Alberto Ferreira de Freitas, Andreza Negreli Santos, Guilherme Canho Bittner, Baltazar Dias Sanabria, Maria Margarida Morena Domingos Levenhagen, Günther Hans-Filho

**Affiliations:** Department of Dermatology, University Hospital Maria Aparecida Pedrossian, Federal University of Mato Grosso do Sul, Medical School, Brazil

## Abstract

**Background:**

To evaluate the surgical treatment results of a consecutive series of patients with nonmelanoma skin cancer in critical facial regions such as the nose, lip, eyelid, ear, forehead, cheek, and chin.

**Methods:**

This was a prospective observational cohort study evaluating the surgical treatment results of 102 patients with nonmelanoma skin cancer who underwent surgical excision and required some type of reconstruction. The reconstruction strategy used, histological type and margins, aesthetic result, and complications were evaluated.

**Results:**

The most common facial site was the nose (48.01%), followed by the eyelid, ear, cheek, forehead, and lip. The most frequently used type of reconstruction was the advancement flap (30.39%), followed by transposition flap (27,45%), rotation flap (14.70%), and grafts (10.78%). Basal cell carcinoma was the most frequent histological type, accounting for 90.19% of the sample, with 54.90% of these cases being of the nodular subtype. Disease-free margins were obtained in 94.11% of the patients, and only one patient presented compromised margins and underwent marginal extension. A good cosmetic result was found in 93.13% of the participants.

**Conclusion:**

Surgical treatment can provide excellent oncological, functional, and cosmetic results in the treatment of patients with nonmelanoma skin cancer at critical facial sites.

## 1. Introduction

Excision of malignant skin lesions located on the face with safe margins and adequate reconstruction can present a challenge to the surgeon [1, 2]. The primary objective is the excision of the lesion with oncological margins to preserve the function of the affected organ while seeking the best possible cosmetic result [2]. Nonmelanoma skin cancer (NMSC) is the most common malignancy in the world [3], and its incidence is increasing [4]. In Brazil, 195,000 new cases were estimated in 2016 [5], with basal cell carcinoma (BCC) being the most frequent, accounting for 75% of all cases [6], followed by squamous cell carcinoma (SCC; 20%) and melanoma (<5%). NMSC affects individuals of all ages but has a higher incidence after the fifth decade of life. Risk factors include sun exposure, particularly in childhood; lighter phototypes; immunosuppression; and genetic predisposition [7]. In addition to surgical excision, there are other therapeutic options such as photodynamic therapy, cryotherapy, radiotherapy, imiquimod, 5-fluorouracil, and intralesional injection of interferon [8, 9]. However, surgical treatment, when possible, yields the lowest recurrence rates and is preferred by most researchers [10]. NMSC is considered to be high risk when located in the ear or central part of the face [7] and may be more difficult to treat when located near the natural head orifices and neck, where resection, margin control, and defect reconstruction may be more difficult [11]. For this reason, research on flaps and grafts for resolving these cases is increasingly encouraged. This study analyzes the surgical strategies used in treating 102 consecutive patients and the initial results obtained.

## 2. Methods

In this prospective cohort study, lesion excision and subsequent surgical reconstruction strategies in 102 consecutive patients with facial BCC and SCC were analyzed, along with the initial histopathological and cosmetic results and possible complications.

Dermatoscopy was used to delimit the lesions and mark the margins: whenever possible minimum width of 5mm and 6mm to BCC sclerodermiform. All patients had previously undergone a biopsy and received a diagnosis of NMSC.

At histopathological analysis, margins larger than 2mm were considered as free, and margins equal to or less than 1mm were considered as coincident and as compromised in cases where there was a lack of margins.

Patients with facial lesions whose treatment required some type of reconstruction with a flap or graft were included in the study. The second-intention wound healing technique was not used in any case.

The research project was submitted to and approved by the Human Research Ethics Committee, and all patients who agreed to participate signed an informed consent form.

## 3. Results

**Figure 1:**
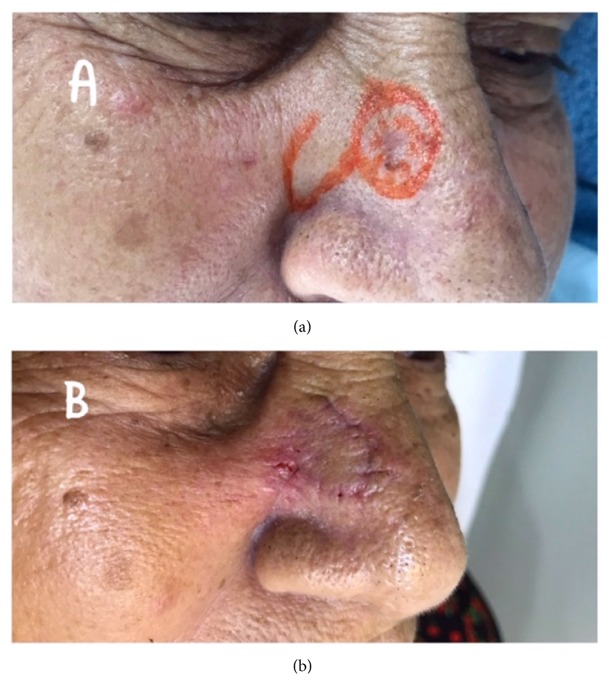
Nasal BCC, after previous marking of dermatoscopy and margin borders (a), and closure with a neighboring flap in transposition. (b) Result with 7 days of evolution.

**Figure 2:**
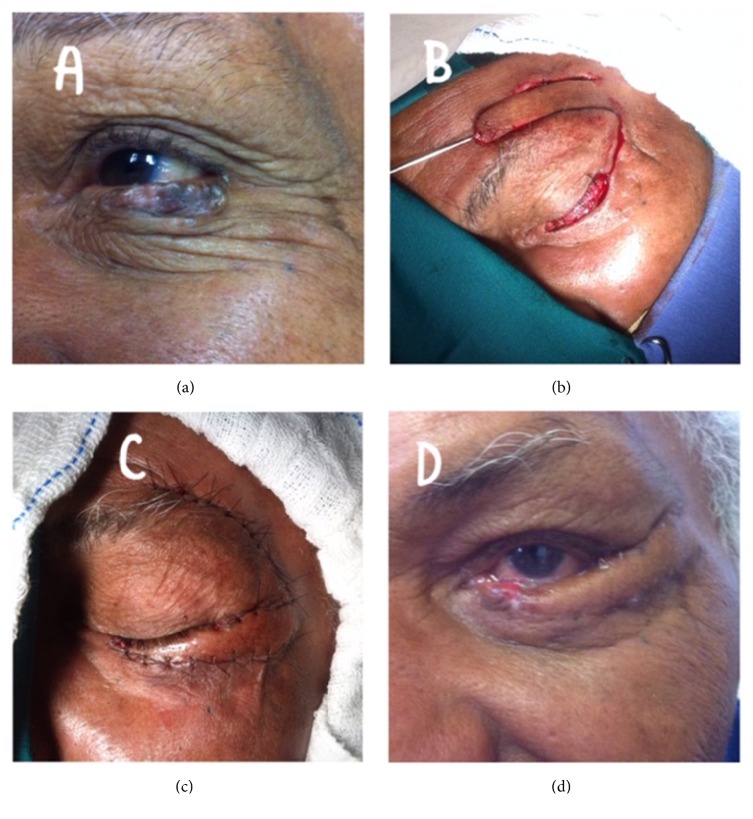
Lower eyelid BCC. (a) Aspect of the lesion. (b) Resection with margin and preparation of the eyebrow flap. (c) Immediate aspect. (d) Aspect with fifteen days of evolution.

**Figure 3:**
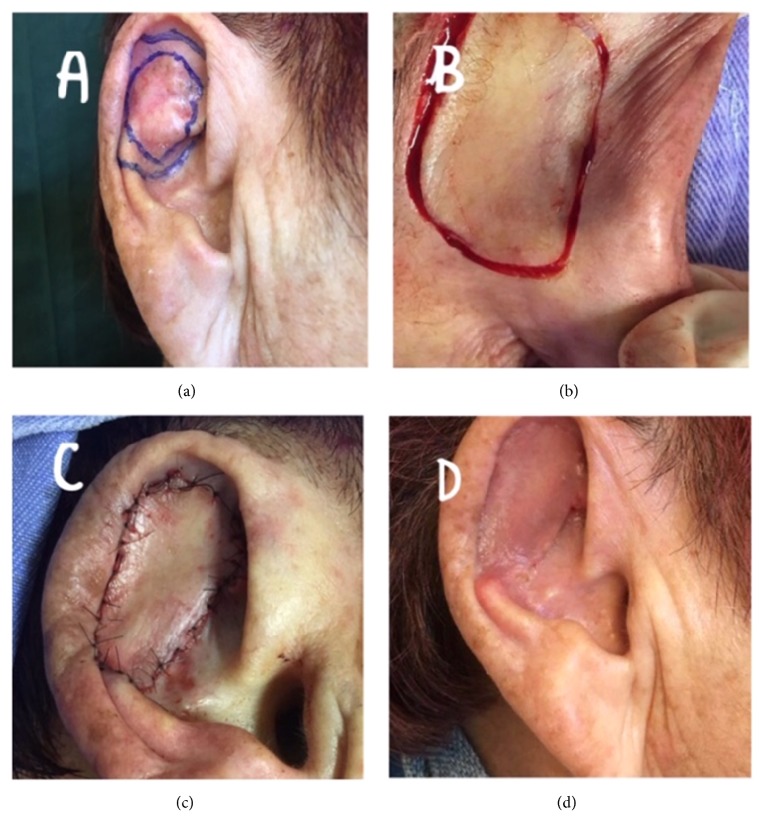
Patient with basal cell carcinoma of the left ear. (a) Boundaries and margins. (b) Retroauricular retail. (c) Immediate result. (d) Final appearance after 30 days.

The group consisted of 102 patients, including 36 (35.29%) men and 66 (64.71%) women, aged between 31 and 96 years (mean age, 69.2 years). Their skin type distribution indicated a predominance of phototypes I to III (82.0%), followed by type IV (18.0%), and no patients had type V. Regarding the most common site on the face, 48.01% of the patients presented lesions on the nose (Figure 1), 17.64% on the eyes (eyelids) (Figure 2), 14.70% on the ear (Figure 3), 7.84% on the forehead, and 7.8% on the cheeks. The remaining 6.66% of the patients presented lesions on the lips and chin. The most commonly used facial reconstruction technique was an advancement flap in 31 patients (30.39%), including 17 island flaps and six Rintala flaps. Transposition flaps were used in 28 patients (27.45%), and rotation flaps were used in 15 patients (14.70%). A partial skin graft was used in 11 patients (10.78%). Regarding the histological type, the majority of the cases were BCC (90.19%), and the most common subtypes were the following: nodular (54.90%), pigmented (16.66%), and sclerodermiform (12.74%). SCC was diagnosed in 6.86% of the patients, with 3.92% and 1.92% of the cases being of grade I and grade II, respectively. Adequate margins were obtained in 94.11% (larger than 2mm) of the patients. Five patients had overlapping margins or margins smaller than 1 mm. These patients were followed up and have not shown signs of recurrence to date. One patient presented compromised margins and was reoperated to extend the margins. None of the patients presented recurrence during the follow-up of two to 36 months. The cosmetic result was considered good and fair in 93.13% and 6.87% of the patients studied, respectively. Results were considered poor by either the research team or patients in presence of unaesthetic scar, retraction, or alteration in organ function or symmetry. The most common complication was partial graft or flap loss, which occurred in 6.86% of the cases. Two patients presented surgical wound infection (1.96%), and two patients presented with scar retraction. All patients were treated with localized care and made satisfactory progress. One patient with nodular BCC on the ear presented moderate bleeding, requiring reoperation to control the bleeding, and progressed well without flap loss.

## 4. Discussion and Conclusions

Surgical treatment of patients with NMSC is preferred by most researchers [10] because it has some advantages over other forms of treatment in terms of treatment time and results achieved. An extensive literature review revealed that surgical excision was the most effective strategy for NMSC treatment [8]. Surgical excision and margin control are the gold standard of BCC treatment [12, 13]. In this series, dermatoscopy was used to define the lesion boundaries, a 5-mm margin whenever possible and 6mm to BCC sclerodermiform. A study recommended the use of dermatoscopy for demarcating the lesion boundaries and stated that the technique can improve the surgical results regarding margin control, especially in places where Mohs surgery is not available [14]. In a reference center for skin cancer treatment, clinical diagnosis, mainly of BCC, aided by dermatoscopy had high accuracy rates [15]. The incidence of compromised margins on the eyelid may reach 39% [6]. In our series, by using dermatoscopy to define the limits of the lesion and margin, the occurrence of compromised margins was very low, even in lesions located on the eyelid. Only one patient presented compromised margins, which represented a good initial oncological result. The use of dermatoscopy apparently aided in the control of surgical margins. The recurrence of BCC on the face when margins are free is very low (<3%) [12]. The cosmetic result was good in most cases. Complications were rare, and patients progressed well with localized care. The use of various types of flaps demonstrates the difficulty of closing defects on the face, which are sometimes small but often near natural and mucous orifices, which always makes margin control and aesthetic and functional closure difficult. The most common histological type is BCC. In this series, its incidence was even higher, approximately 90%, than the mean incidence in the literature (75%) [6]. In an extensive sample of 500 patients with head and neck NMSC, a study revealed a BCC incidence of 72% and SCC incidence of 28% [16].

Mohs technique is considered ideal to preserve normal tissue, being thus most recommended to the head and neck nonmelanoma skin cancer treatment. Besides, it allows better three-dimensional control of deep margins. When it is not available, macroscopic margins control can be performed by beginning resection at lateral margins leaving the deep one in central part of lesion last. The presence of compromised margins requires immediate surgical reassessment [17].

Treatment of NMSC on the face, at critical sites such as the eyelids, nose, ears, forehead, lips, and chin, is a challenge to surgeons and is based on achieving the best oncological, functional, and cosmetic results [2]. Surgery and adequate surgical reconstruction are an ideal treatment modality and may yield good results, as demonstrated in this consecutive series of treated patients. Dermatoscopy aids in the clinical diagnosis and margin control in BCC.

## Data Availability

The data used to support the findings of this study are included within the article.
